# Communal Living by Bacteria and the Pathogenesis of Urinary Tract Infections

**DOI:** 10.1371/journal.pmed.0040349

**Published:** 2007-12-18

**Authors:** Steven M Opal

## Abstract

Steven Opal reviews the phenomenon of bacterial communities and discusses the role played by bacterial communication and cooperation in host-pathogen interactions, particularly in urinary tract infection.

In this issue of *PLoS Medicine*, Rosen and colleagues [1] present convincing evidence that intracellular communities of Escherichia coli commonly exist and are likely of to be of clinical significance in uncomplicated bacterial cystitis. This novel finding is remarkable in many respects, not the least of which is the circuitous route by which our understanding about bacterial communities has evolved in the pathogenesis of infectious diseases.

Since the inception of the germ theory of disease over 150 years ago, it has generally been assumed that potential pathogens invade the host essentially as solitary microorganisms (“lone soldiers”). Successful pathogens must find a susceptible host and then gain access to host tissues through a defect in epithelial barriers. The pathogen must replicate rapidly, and either overwhelms the host's innate and adaptive immune system, or successfully evades antimicrobial defenses by avoiding host recognition and clearance mechanisms [2].

This traditional view of microbial pathogenesis was turned on its head about 30 years ago when three microbiologists, Nealson, Platt, and Hastings [3], made the seemingly innocuous discovery that some marine bacteria actually have the capacity to communicate with each other and coordinate their activities to their mutual benefit. They investigated a halophilic (see Glossary) bacterium known as Vibrio fischeri, which forms an unusual symbiotic relationship with the Hawaiian bobtail squid (Euprymna scolopes). The bioluminescent V. fischeri is taken up by light organs along the body of the squid and, when high concentrations of bacteria are attained, the bacterium induces its luciferase genes to generate visible light.

**Linked Research Article**

This Research in Translation article discusses the following new study published in *PLoS Medicine*:

Rosen DA, Hooton TM, Stamm WE, Humphrey PA, Hultgren SJ (2007) Detection of intracellular bacterial communities in human urinary tract infection. PLoS Med 4(12): e329. doi:10.1371/journal.pmed.0040329

Analyzing urine specimens from women with bladder infections, David Rosen and colleagues find evidence for intracellular bacterial communities, which have been associated with recurrent urinary tract infections in mice.

The bacteria benefit from this mutualistic relationship, as the squid provides a source of nutrients for the bacteria. Meanwhile, the squid benefits from the microbial light source, which provides a unique form of camouflage for the squid during nighttime mating rituals. The adult squid populations congregate in large numbers along the water's surface for mating. On moonlit nights, the squid's body is silhouetted against a starlit sky and is thus readily detectable from below by predatory fish. Light organs on the underside of the squid provide a “starry sky camouflage” by their association with the light-generating clusters of V. fischeri.

The bioluminescent potential of this Vibrio species is only activated with high bacterial densities. But how do individual bacterial cells know their relative concentration within the light organ of the squid? The answer was discovered when V. fischeri was found to produce a soluble “quorum sensing” molecule that co-activates the operon for luciferase production in neighboring bacteria when the organisms are in high concentration [3].

## Bacterial Communities, Communication, and Cooperation in Pathogenesis

This observation was initially viewed as a mere curiosity unique to marine microbiology until genome searches revealed homologous quorum sensing genes among many clinically relevant microbial pathogens [4–8]. Remarkably, many bacterial pathogens, including E. coli, use quorum sensing genes to regulate critically important virulence genes during microbial invasion.

At least three separate systems of quorum sensing and auto-induction molecules exist in bacteria. The first is highly homologous to the system found in V. fischeri and uses acyl homoserine lactone (acyl HSL) molecules for signaling. This system is widely used by medically important gram-negative bacterial pathogens. The second system, found in gram-positive bacteria, is a functionally homologous version of quorum sensing that uses a series of short cyclic peptides and a two-component receptor–kinase signaling pathway. A third hybrid system, found in both gram-positive and gram-negative bacterial species, uses some elements similar to the acyl HSL system of gram-negative bacteria and the receptor–kinase system of gram-positive bacteria [4,6].

What is now clear is that bacterial communication and cooperation is a ubiquitous phenomenon and that these communications systems are control elements in host–pathogen interactions [4]. The entire complement of genes that contribute to virulence in bacterial pathogens is known as the virulome. Quorum sensing molecules are a major regulator of the virulome [5]. Up to 15% of the open reading frames of bacteria is controlled by quorum sensing molecules [9]. Quorum sensing can promote the growth of related strains of bacteria and simultaneously inhibit the growth of other bacterial [10] or even fungal [9] organisms competing for the same ecologic niche. Critical virulence determinants such as toxin production, sporulation, plasmid transfer, invasion gene synthesis, and various immune evasion mechanisms of bacteria are controlled by quorum sensing genes.

Recent evidence now reveals that these communication pathways can cross kingdom boundaries [9]. Bacterial acyl HSL molecules can alter transcriptional programs in human cells and this system may be used to subvert the host defenses during microbial invasion. The quorum sensing system of Pseudomonas aeruginosa can sense human gamma interferon, alerting the bacterial pathogen to physiologic stress within the infected host [11].

## Biofilms and Bacterial Communal Living

Quorum sensing is essential to the production of healthy and fully developed biofilms. These palisade-like complex multicellular structures are relatively stable communities of bacterial populations living in a sessile, protected environment [12]. Their slow rate of metabolism and their physical location within biofilm exopolysaccharide capsules protect bacteria against the bactericidal effect of antibiotics and host clearance by opsonins and neutrophils. A major survival advantage is gained if bacterial populations can cooperate and live in a protected, communal setting within the human host [13,14].

Biofilm communities develop rapidly on catheter surfaces and on mucous membranes along epithelial surfaces. Bacterial communities residing on urinary catheter surfaces are relatively refractory to the lytic action of many antibiotics. This resistance undermines the capacity of antimicrobial agents to eradicate infections on biofilmladen foreign bodies within the genitourinary system [12–14].

**Five Key Papers in the Field**

**Qazi et al., 2006** [10] The same quorum sensing molecules that promote growth in gram-negative bacterial pathogens inhibit the growth of S. aureus; this pathway could favor the quorum sensing gram-negative pathogens over S. aureus when in competition for limited resources during microbial invasion of the host.

**Shiner et al., 2005** [9] This manuscript presents surprising evidence of direct communication between bacterial quorum sensing molecules and human transcriptional signaling induced by bacterial pathogens.

**Wu et al., 2005** [11] P. aeruginosa uses its quorum sensing apparatus to detect and signal transcriptional programs for virulence expression in response to human interferon gamma. This interferon marker signals an inflammatory stress in the host during microbial invasion by the microbial pathogen.

**Novick et al., 2003** [5] An excellent review of the sophisticated control of staphylococcal virulence and the regulation of the virulome of this gram-positive pathogen by its quorum sensing and auto-induction peptides.

**Smith et al., 2003** [8] This paper provides a detailed description of the dual quorum sensing systems of P. aeruginosa and their respective roles in biofilm formation and bacterial pathogenesis.

Rosen and colleagues [1] now go one step further, showing that intracellular communities of gram-negative bacteria are a widespread finding even in uncomplicated lower urinary tract infections (UTIs). They detect intracellular bacterial communities (IBCs) in 18% of young, sexually active women with E. coli cystitis. Up to 41% of urine specimens in women with cystitis have evidence of filamentous bacterial forms, a morphologic hallmark of recent residence among sessile bacterial communities. Similar findings have previously been reported in the murine model of cystitis [15]. Cytologic evidence of IBCs within exfoliated urinary epithelial cells is readily demonstrable in women with acute cystitis. Immunofluorescence studies and electron microscopy confirms that these large bacterial communities exist inside facet cells lining the transitional epithelium of the urinary bladder.

**Glossary**

**Acyl homoserine lactone:** a highly soluble, cyclic homoserine moiety covalently linked to short fatty acids that functions as a quorum sensing signal molecule for many gram-negative bacteria

**Auto-induction molecule:** diffusible low-molecular weight signal molecule recognized by the microorganism that produced the molecule and capable of inducing changes in the transcription of specific genes

**Halophilic:** capable of living in a salty environment

**Open reading frames:** Sequences of DNA that include the start codon AUG and appropriate translation sequences that generate a functional protein product

**Operon:** a series of adjacent open reading frames transcribed from a single promoter site that generates gene products working on the same metabolic pathway

**Quorum sensing:** ability to detect the density of a bacterial population and alter genetic programs in response to that population density

**Receptor–kinase signaling pathway:** two-component system consisting of a membrane receptor to detect extracellular ligands, linked to an intracellular phosphorylating enzyme, which alters the transcriptional programs of the bacterial cell

**Toll-like receptor:** Pattern recognition receptor used by the innate immune system to detect and respond to highly conserved microbial mediators such as bacterial lipopolysaccharide or bacterial flagellin

Uropathogenic E. coli have the capacity to invade epithelial lining cells, where they find sanctuary from immune surveillance and urinary clearance mechanisms [15,16]. The intracellular environment shelters bacteria from many antimicrobial agents that are primarily restricted to the extracellular space. The presence of large bacterial communities living as relatively quiescent, filamentous bacteria inside epithelial cells has not been described previously in women with uncomplicated lower urinary tract infections.

## Future Research Priorities

There are numerous implications of these findings for the pathogenesis of urinary tract infections, and for the possible treatment options available to prevent recurrent infection in UTI-prone women. These sessile bacterial communities might easily provide a sequestered site from antimicrobial defenses, allowing for repopulation of the urinary tract after a seemingly appropriate course of antimicrobial agents for bacterial cystitis. The formation of IBCs is a Toll-like receptor (TLR4)-dependent process in the murine system [17] and is likely to be so in humans as well. A large number of common polymorphisms exist in the TLR4 signaling apparatus in humans that affect the transcriptional rates of acute phase response genes [18–20]. What relationship exists between the frequency of IBCs, susceptibility to recurrent urinary tract infections, and human TLR4 signaling polymorphisms [20]?

The current study was performed in young, sexually active women. Many women had a history of recurrent bacterial cystitis. What is the prevalence of IBCs in children, the elderly, or men or women experiencing their first episode of UTI? Does the presence of IBCs in first episodes of UTI indicate a greater propensity for treatment failure or relapsing urinary tract infection? What is the prevalence of IBCs in patients with upper urinary tract infections or complicated urinary tract infections associated with urinary obstruction or foreign bodies? Are similar IBCs identifiable with pathogens other than E. coli? Staphylococcus aureus regulates many of its virulome invasion genes through its auto-inducing peptides and quorum sensing system [4,5]. It would be of great interest to determine if urinary tract colonization or infection by methicillin-resistant S. aureus or other gram-positive bacterial pathogens use communal intracellular invasion strategies.

The findings of Rosen et al. [1] raise the intriguing possibility that detection of these relatively quiescent IBCs might help identify patients who may benefit from a longer course of antimicrobial agents, or from agents that penetrate the intracellular space. Inhibitors of quorum sensing have already been shown to interfere with biofilm formation and to limit the virulence potential of some bacterial pathogens [21–24]. Perhaps inhibitors of quorum sensing and biofilm formation could prevent relapse from urinary tract infections. The current study indicates that urinary tract infections will be a fertile area for future research in the field of bacterial communities, biofilm formation, and microbial pathogenesis.

**Figure 1:**
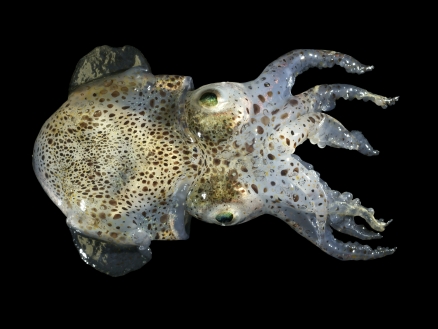
Bobtail squid have a symbiotic relationship with bioluminescent bacteria (Vibrio fischeri), which inhabit a special light organ in the squid's mantle. (Photographer: Hans Hillewaert, marine biologist and one of the administrators of Wikispecies [http://species.wikimedia.org/wiki/User:Lycaon])

## Abbreviations

acyl HSL: acyl homoserine lactone
IBC: intracellular bacterial community
TLR4: Toll-like receptor
UTI: urinary tract infection
